# Bioactive Constituents and Toxicological Evaluation of Selected Antidiabetic Medicinal Plants of Saudi Arabia

**DOI:** 10.1155/2022/7123521

**Published:** 2022-01-17

**Authors:** Ali S. Alqahtani, Riaz Ullah, Abdelaaty A. Shahat

**Affiliations:** ^1^Department of Pharmacognosy, College of Pharmacy, King Saud University, Riyadh, Saudi Arabia; ^2^Medicinal Aromatic and Poisonous Plants Research Centre, College of Pharmacy, King Saud University, Riyadh, Saudi Arabia

## Abstract

The purpose of this review is to summarize the available antidiabetic medicinal plants in the Kingdom of Saudi Arabia with its phytoconstituents and toxicological findings supporting by the latest literature. Required data about medicinal plants having antidiabetic activities and growing in the Kingdom of Saudi Arabia were searched/collected from the online databases including Wiley, Google, PubMed, Google Scholar, ScienceDirect, and Scopus. Keywords used in search are in vivo antidiabetic activities, flora of Saudi Arabia, active ingredients, toxicological evaluations, and medicinal plants. A total of 50 plant species belonging to 27 families were found in the flora of Saudi Arabia. Dominant family was found Lamiaceae with 5 species (highest) followed by Moraceae with 4 species. *β*-Amyrin, *β*-sitosterol, stigmasterol, oleanolic acid, ursolic acid, rutin, chlorogenic acid, quercetin, and kaempferol are the very common bioactive constituents of these selected plant species. This paper has presented a list of antidiabetic plants used in the treatment of diabetes mellitus. Bioactive antidiabetic phytoconstituents which showed that these plants have hypoglycemic effects and highly recommended for further pharmacological purposes and to isolate/identify antidiabetes mellitus (anti-DM) active agents also need to investigate the side effects of active ingredients.

## 1. Introduction

Medicinal plants are used for the treatment of different infections [1, 2]. These plants contributed as a source of inspiration for novel therapeutic compounds [3]. The medicinal value of plants is due to the presence of a wide variety of secondary metabolites including alkaloids, glycosides, tannins, volatile oil, and terpenoids [4, 5]. Medicinal plants and their extracts represent a rich source of crude medications that possess therapeutic properties. Indeed, the World Health Organization reports that various plant fractions and their dynamic constituents are utilized as traditional medicines by 80% of the world population [6]. Plants are the primary source for different pharmaceutical, perfumery, flavor, and cosmetics industries; the use of modern drugs dramatically resulted into resistant microorganisms toward different modern drugs; the researchers are now in search for alternate source of treatment of various disorders [7, 8]. For this purpose, the medicinal herbs are the best alternate to various drugs. Most of natural products possess interesting biological activities and medicinal potential. Various herbs, fruits, and grains have been found to have different important biological activities such as antioxidant, [9] antitumor, antimutagenic, antidiabetes, antianalgesic, [10] antidementia, inflammation inhibitory effect, [9] antitumor, [11] anticancer, [12] antimicrobial, antileishmanial, and antimalarial properties [13, 14]. The consumption of natural antioxidants will reduce risk of many diseases including cancer, cardiovascular disease, diabetes, and other diseases allied with aging [15]. For natural antioxidants, a larger number of medicinal herbs have been evaluated by applying laboratories' developed procedures. Plants derived substances, collectively called phytonutrients or phytochemicals, been recognized as good source of natural antioxidants [16, 17].

The Kingdom of Saudi Arabia is a huge arid land with an area of about 2,250,000 km^2^ covering the major part of the Arabian Peninsula, characterized by different ecosystems and diversity of plant species. The climate in Saudi Arabia differs greatly between the coast and the interior. High humidity coupled with more moderate temperatures is prevalent along the coast, whereas aridity and extreme temperatures characterize the interior. The flora of Saudi Arabia is one of the richest biodiversities in the Arabian Peninsula and comprises very important genetic resources of crops and medicinal plants. Saudi Arabia contains 97 trees, 564 shrubs, and about 1620 herbs, which cover, respectively, 4.25%, 24.73%, and 71.02% of higher plant diversity of the country [18].

Diabetes mellitus is one of the most prevalent diseases in endocrine gland system with an increasing incidence in human community [19]. Type I diabetes is caused by insulin secretion deficit, while type II diabetes is accompanied with progressive rate of insulin resistance in liver and peripheral tissues, reducing *β*-cell mass, and deficient insulin secretion [20, 21]. This disease brings about acute metabolic side effects including ketoacidosis, hyperosmolar coma accompanied with chronic disorders, and long term, adverse side effects such as retinopathy, renal failure, neuropathy, skin complications, as well as increasing cardiovascular complication risks [22, 23]. Also, common symptoms of diabetes are frequent urine, thirsty, and overeating [24]. Diabetes inflicts 100 million people yearly and is recognized as the seventh cause of death in the world [25]. It has been estimated that the number of diabetic people will increase from 150 million individuals in 2003 to 300 million by 2025 [26]. The essential and effective drugs for diabetes mellitus are insulin injection and hypoglycemic agents, but these compounds possess several adverse effects and have no effects on diabetes complications in long term. Therefore, it is important to find effective compounds with lower side effects in treating diabetes [27]. Medicinal plants are good sources as alternative or complementary treatments for this and other diseases [28–30]. Although various plants have been traditionally used throughout history to reduce blood glucose and improve diabetes complications, there is not enough scientific information about some of them. Herbal medicines are commonly prescribed throughout the world because of low side effects, availability, roughly low cost, and also its effectiveness [31, 32].

In Saudi Arabia, the number of people who suffer from DM increased from 890,000 in 2000 to a staggering projection of 2,523,000 in 2030. In 2011, Saudi Arabia reported a prevalence of DM at 30% of the total population, with a rate of 27.6% in women and 34.1% in men [33]. According to 2010 data from several sources (WHO, World Bank, UNESCO, CIA, and individual country databases), DM is the number three disease-related cause of death in Saudi Arabia [34].

In the present situation, herbal medicines' usage has significantly increased and published studies from developed countries emphasize that a paramount proportion of medicines supplied by them have herbal origins, so growing and producing the herbal medicines could be helpful to both economic development and community's health [35]. Keeping in mind the importance of medicinal plants, in the current review various medicinal plants used for antidiabetic treatment around the world, native to or cultivated in Saudi Arabia, are documented for the purpose to provide up-to-date insight on medicinal plant used for DM, so that researcher easily selects plant for bioscreening and active constituents' identification purposes. Therefore, we invite researchers' attention to carry out detailed ethnopharmacological and toxicological studies on unexplored antidiabetic plants in order to provide reliable knowledge to the patients and develop novel antidiabetic drugs.

## 2. Methods

Required data about medicinal plants having antidiabetic activities and growing in the Kingdom of Saudi Arabia were searched/collected from the online databases including Wiley, Google, PubMed, Google Scholar, ScienceDirect, and Scopus. Keywords used in search are in vivo antidiabetic activities, flora of Saudi Arabia, active ingredients, toxicological evaluations, and medicinal plants. Latest published data approximately in the last ten years with the key outcome of change in blood glucose level in animal model were included. One or two articles are selected as references for each plant's species on priority basis from the journals found in web of science and latest years.

## 3. Results

The names, families, used parts, location, and antidiabetic properties in animal model of the native/cultivated Saudi medicinal plants are summarized in Table 1. The active ingredients and toxicological effect of these plants in animal model are given in Table 2. A total of 50 plant species belong from 27 families were found in the flora of Saudi Arabia. Dominant family was found Lamiaceae with 5 species (highest) followed by Moraceae with 4 species.

## 4. Discussion

The majority of the experiments confirmed the benefits of medicinal plants with hypoglycemic effects in the management of diabetes mellitus. From Table 1, it can be concluded that among the plants used for the treatment of diabetes, *H. salicornicum, T. oliverianum, A. cepa, A. herba-alba, Teucrium polium, Sesamum indicum, Z. spina-christi,* and *U. dioica* seem to be most common plants used to treat diabetes and are available everywhere in the world. The leaves were most commonly used plant part, and other parts (root, stem, bark, flower, seed, and whole plant) were also useful for curing. The most common diabetic model that was used was the streptozotocin and alloxan-induced diabetic mouse or rat as diabetic models. The most commonly involved active constituents are flavonoid, alkaloid, saponin, carbohydrate, vitamins, amino acid and its derivatives, phenol and its derivatives, and benzoic acid derivatives. The very common phytoconstituents, *targeted metabolic pathways,* and its structure are given in Table 3 [194, 195]. The native to or cultivated plant species of the kingdom given in Table 1 are selected from the published literature about ethnobotanical value and antidiabetic potential of medicinal plants around the world. The ethnobotanical information reports about 800 plants that may possess antidiabetic potential [196, 197]. Jeeva and Anlin also reported 177 plants belonging to 156 genera and 76 families used traditionally for antidiabetic treatment [198]. In the Middle East countries, there are 129 plant species still in use in traditional Arabic medicine. This indicates that the medicinal plant species require preservation as well as the ethnobotanical and ethnopharmacological knowledge. The preservation of the herbs is an essential requirement for maintaining traditional Arabic medicine as a medicinal and cultural resource [199]. The selected plant species *H. salicornicum, T. oliverianum, A. cepa, A. herba-alba, Teucrium polium, Sesamum indicum, Z. spina-christi,* and F. religiosa are the native Saudi medicinal plants traditionally used for the treatment of DM [200]. Similarly published data showed that 20 medicinal plants are traditionally used in Tabuk region of Saudi Arabia [201]. *Anisotes trisulcus, Artemisia judaica,* and *Moringa peregrine* are used in Al Khobah village, Saudi Arabia, for DM treatment [202]. *O. europaea* is used in Al Bahah region of KSA for DM treatment [203]. *C. roseus, A. cepa, U. dioica, A. aspera, C. intybus, C. cyminum, F. bengalensis, C. colocynthis,* and *T. polium* are the highly investigated medicinal plants for antidiabetic potential [204–206].

Desiring to contribute to the conservation priorities of traditional medicine knowledge of various medicinal plants native to or cultivated in Saudi Arabia and to make it easy and familiarized with disease treatment, the present compilation was conducted. According to the International Union for Conservation of Nature and the World Wildlife Fund, there about 15,000 medicinal plant species are threatened with extinction from overharvesting and habitat destruction and 20% of their wild resources have already been nearly exhausted with the increasing human population and plant consumption [207]. Each plant species lost due to extinction phenomena could represent not only the loss of healthcare saving cures for special diseases but also the loss of probable primary metabolite liker protein- or vitamin-rich foods [208]. Medicinal plants have been cited as a potential source of heavy metal toxicity to both man and animals. The most common heavy metals implicated in human toxicity include lead, mercury, arsenic, and cadmium, although aluminum and cobalt may also cause toxicity. From the study, the levels of these metals differed in the same plant collected from different geographical locations. A study conducted showed that the levels of lead in Cassia alata varied from 17.7 to 4.45 *μ*g/g for the 5 collection sites. Similarly for Cassia occidentalis and Rauvolfia vomitoria, the level is varied between 7.85-4.35 and 9.25–1.55 *μ*g/g, respectively. Similarly, that of aluminum varied between 105.53 and 23.3 for Rauvolfia vomitoria and 104.25–12.4 *μ*g/g for Paullinia pinnata. The levels of heavy metals also varied for different plants collected from the same location. Uptake of metals by plants is influenced by a number of factors including metal concentrations in soils, cation exchange capacity, soil pH, organic matter content, types and varieties of plants, and plant age. However, the prevailing factor is the concentration of the metal in the soil and thus the existing environmental conditions [209]. Another study conducted on onion bulb showed that the concentrations of Cr in onion bulb and Fe in onion leaf were above the permissible level (2.3 mg/kg, 425.5 mg/kg) set by FAO/WHO at Mojo (4.87 mg/kg, 1090.40 mg/kg), Meki (4.13 mg/kg, 1836.47 mg/kg), and Ziway (3.33 mg/kg, 764.33 mg/kg), respectively. The results generally indicate that the consumption of these onion bulbs could be the health risk respective to Cr [210]. Therefore, it is suggested that the medicinal plant source for the treatment of diabetes must not be taken from heavy metal contaminated areas to avoid their uptake by the plants because migration of these contaminants into noncontaminated areas (or leaching through the soil and spreading of heavy metal contaminated sewage sludge) are a few examples of events contributing to contamination of the ecosystem.

## 5. Conclusion and Recommendations

The present review provides a picture of medicinal plants that have been studied as anti-DM drugs, which can be grown either in combination with other medicinal plants or alone as treatment for diabetes and drawbacks should be properly addressed so that medicinal plants can be effectively utilized as anti-DM drugs. Diabetes is a metabolic disorder which can be considered as a major cause of high economic loss which can in turn impede the development of nations. Moreover, uncontrolled diabetes leads to many chronic complications such as blindness, heart failure, and renal failure. In order to prevent this alarming health problem, the development of research into new hypoglycemic and potentially antidiabetic agents is of great interest. In conclusion, this paper has presented a list of anti-DM plants used in the treatment of diabetes mellitus. Bioactive antidiabetic phytoconstituents which showed that these plants have hypoglycemic effects and highly recommended for further pharmacological purposes and to isolate/identify anti-DM active agents also need to investigate the side effects of active ingredients.

## Figures and Tables

**Table 1:** Antidiabetic medicinal plants growing in Saudi Arabia.

S. no.	Names of plants	Family	Part used	location	Antidiabetes Activities
1.	*Allium cepa*	Liliaceae	Bulb	Central Saudi Arabia [36]	Ethanol extract of *A. cepa* in STZ-induced diabetic rats causes 66% decreased at 200 mg/kg after 24 h in blood glucose level [37]. 0.4 g/100gbw of A. cepa reduced 50% the fasting glucose levels of diabetic rats [38]. Similar results reported by other researchers [39].
2.	*Anthemis herba-alba*	Compositae*/*Asteraceae	Aerial parts	Farasan Island of Red Sea [40]	72% plasma glucose levels decreased in albino mice by ethyl alcohol extract of *Artemisia herba-alba* [41]
3.	*Cichorium intybus*	Asteraceae	Seeds	Qassim region [42]	C. intybus leaf powder, ethanol, aqueous seed extracts, and hexane extracts led to a decrease in blood glucose levels to near normal value. Hypoglycemic effects of *C. intybus* were observed in diabetic rats, and a dose of 125 mg of plant extract/kg body weight exhibited the most potent hypoglycemic effect [43–45]
4.	*Clitoria ternatea*	Fabaceae	Aerial parts	Cultivated throughout Saudi Arabia [46]	The aqueous extract of *Clitoria ternatea* leaves and flower administered for 84 days to diabetic rats significantly decreased blood glucose [46–48]
5.	*Ficus carica*	Moraceae	Leaves	Southwest of Saudi Arabia [49]	Different extracts and fractions of *F. carica* showed a clear hypoglycemic effect in diabetic rats. *F. carica* leaves exerted significant effect on carbohydrate metabolism enzymes with promising hypoglycemic and hypolipidemic activities in type 2 diabetic rats [50, 51]
6.	Ficus benghalensis	Moraceae	Bark	Riyadh [52]	In streptozotocin-induced diabetic rats, bark aqueous extract, and an isolated compound, *α*-amyrin acetate exhibited antidiabetic activity by decreasing the blood glucose level and increasing the HDL level [53]
7.	*Ficus religiosa*	Moraceae	Root bark, stem bark, aerial roots	Riyadh [52]	The aqueous extract of bark and ethanol extract of leaves and fruits had a promising antidiabetic effect in streptozotocin-induced diabetic rats by decreasing the blood glucose, serum triglyceride, and total cholesterol levels and increasing serum insulin, body weight, and glycogen content in the liver and skeletal muscle [53]
8.	*Ficus microcarpa*	Moraceae	Leaves	Riyadh [52]	*F. microcarpa* leaves showed protective effect against alloxan-induced diabetic rats by reducing blood glucose, cholesterol and triglyceride levels, and increased insulin level [53]
9.	*Hypericum perforatum*	Hypericaceae	Leaves	Western Saudi Arabia [54]	H. perforatum ethyl acetate extract possesses potent antihyperglycemic activity in STZ-induced diabetic rats [55].
10	*Anethum graveolens*	Apiaceae	Seeds	Makka [56]	Different extracts and tablets of *Anethum graveolens* possess potent antihyperglycemic activity in alloxan-induced diabetic mice [57]
11	*Cuminum cyminum* L.	Apiaceae or Umbelliferae	Seeds	Makka [56]	Oral administration of cumin seeds crude ethanol extract and glibenclamide to *diabetic rats* significantly and progressively restored toward normal. Cumin seeds crude ethanol extract and glibenclamide reduced *plasma glucose* levels by 38.34 and 37.73%, respectively, compared with diabetic control [58]. Other studies also reported similar results [59].
12	*Marrubium vulgare*	Lamiaceae	Whole plant	Widely distributed in Saudi Arabia [60]	M. vulgare extracts lower blood glucose level 30 to 60% in dose-dependent manner in streptozotocin-induced diabetic rats [60].
13	*Mentha longifolia*	Lamiaceae	Whole plant	Madinah [61]	Remarkable antidiabetic, anticholinesterase, and antityrosinase effects were recorded for the mint oil [61, 62]. Still need to investigate in vivo antidiabetic potential.
14	*Origanum syriacum*	Lamiaceae	Leaves	Saudi Desert [63]	The whole plant extract of *O. syriacum* at 100 and 400 mg/kg significantly lowers glucose level in diabetic induced rats [64].
15	*Teucrium oliverianum*	Lamiaceae	Aerial parts	Throughout Saudi Arabia [65,66]	Aqueous and ethanol extract of Teucrium oliverianum were tested for antidiabetic activity in alloxan-induced diabetic mice. Both extracts significantly reduced blood sugar levels [65]
16	*Teucrium polium*	Lamiaceae	Leaves	Madinah [67]	Infusion orally (64% decrease glucose level) and intraperitoneal of different extracts of *T. polium* caused significant reductions in blood glucose concentration in STZ hyperglycemic rats [68]
17	*Achyranthes aspera*	Amaranthaceae	Whole plant	Al Hada Road Taif [69]	The methanolic and ethanolic extract of A. aspera exhibited significant hypoglycemic activity in streptozotocin-induced diabetic rats [70]
18	*Aerva lanata*	Amaranthaceae	Leaves	Southwest region of Saudi Arabia [71, 72]	Extracts of *Aerva lanata* and glibenclamide were found to significantly (*P* < 0.01 and *P* < 0.05) reduce the blood glucose level and lipid profile in streptozotocin-induced diabetic rats [73]
19	*Alternanthera sessilis*	Amaranthaceae	Whole plant	Hail region, Saudi Arabia [74]	In diabetic mice at doses of 50, 100, 200, and 400 mg per kg body weight, the extract reduced blood sugar levels by 22.9, 30.7, 45.4, and 46.1%, respectively, compared to control animals. By comparison, a standard antihyperglycemic drug, glibenclamide, when administered at a dose of 10 mg per kg body weight, reduced blood glucose level by 48.9% [75]
20	*Carissa edulis*	Apocynaceae	Leaves	Southern region of Saudi Arabia [76]	Oral administration of *C. edulis* extracts of the leaves significantly reduced the blood glucose level in STZ diabetic rats [77].
21	*Catharanthus roseus*	Apocynaceae	Flower, leaves, stem, and root	Western Saudi Arabia [78]	C. roseus (100 mg/kg BW) lowered the glucose level more than metformin-treated group (100 mg/kg BW) in STZ-induced hyperglycemia rats. C. roseus 200 mg/kg dose was found to be more effective in reducing fasting blood glucose levels [79]
22	*Rhazya stricta*	Apocynaceae	Leaves, seeds	Middle and western region of Saudi Arabia [80]	Extracts *Rhazya stricta* lowered 37.9% blood glucose level in the streptozotocin-induced diabetic rats. Serum cholesterol and triglyceride levels were significantly (*P* < 0.05) reduced in the treated diabetic group compared to the untreated diabetic group [81]
23	*Calotropis procera*	Asclepiadaceae	Latex	Al-Kharj [82]	Different extracts of *C. procera* at dose of 250 mg/kg were orally administered as single dose per day to diabetes-induced rats for the period of 15 days significantly decreases blood glucose level to the level of standard drug glibenclamide [83]
24	*Opuntia dillenii*	Cactaceae	Fruit	Jazan Region [84]	Researcher observed the significant hypoglycemic activity of Opuntia dillenii extract in streptozotocin-induced diabetic mice and rabbits [85]
25	*Opuntia ficus-indica*	Cactaceae	Stem	Jazan Region [84]	Powder and water extract of *O. ficus-indica* significantly (in comparison with control group) returned blood glucose level to the initial level, 180 min after administration in STZ-induced diabetic rats [86]. Many studies confirmed the hypoglycemic activities of *O. ficus-indica* [87]
26	*Capparis decidua*	Capparaceae	Fruits, seeds	Jazan Region [84]	*C. decidua* extracts at dose level of 200 and 800 mg/kg significantly reduce sugar level (in a dose-dependent manner) compared to standard drug in STZ-induced diabetic and normal rats [88].
27	*Beta vulgaris*	Chenopodiaceae	Root bark	North Hejaz and Eastern Najd region of Saudi Arabia [89]	Extract of *B. vulgaris* at does level 50, 100, and 200 mg/kg of significantly reduced sugar level and increased in insulin level (in a dose-dependent manner) in streptozotocin or alloxan-induced diabetic mice [90]. Other researchers also concluded similar finding in STZ-induced diabetic rats [91].
28	*Haloxylon salicornicum Bunge*	Chenopodiaceae	Whole plant	Wadi-Hafr-Al-Batin, Saudi Arabia [92]	Ethanol extract (100 and 200 mg/kg of bw) of *H. salicornicum* (oral administration) exhibited persistent hypoglycemic effects in STZ-induced diabetic rats [93]
29	*Evolvulus alsinoides*	Convolvulaceae	Whole plant	Jazan Region [84]	*E. alsinoides* ethanol extract at dose level (150 mg/kg bw) in normal and streptozotocin-induced diabetic rats leads to hyperglycemia in experimental diabetic rats that decreased utilization of glucose by insulin-dependent pathways [94, 95]
30	*Ipomea aquatica*	Convolvulaceae	Whole plant	Jazan Region [96]	*I. aquatica* ethanol extract at dose level (10, 100, and 1000 *µ*g/ml in streptozotocin-induced diabetic rats significantly (*P* < .05) exhibited the ability to enhance insulin-mediated glucose uptake into 3T3F442A adipocytes cells compared to insulin alone [97]. Another study confirmed that doses (200 mg/kg and 400 mg/kg) reduced blood glucose level, and it was statistically highly significant (*P* < 0.001) in comparison with control group [98].
31	*Citrullus colocynthis*	Cucurbitaceae	Fruits	Jazan Region [84]	1 ml/kg and 2 ml/kg of *C. colocynthis* extract (orally administered) stabilized animal body weight and ameliorated hyperglycemia in a dose- and time-dependent manner in alloxan-induced diabetic rats [99]
32	*Citrullus lanatus*	Cucurbitaceae	Seed	Wadi Lajab, Saudi Arabia [100]	*C. lanatus* seed extract (2, 4 g/kg) treatment significantly lowers glucose level which suggested that *C. lanatus* had antidiabetic property in STZ-induced diabetes mice [101]. Other researcher also concluded similar finding in STZ-induced diabetic rats [102]
33	Coccinia grandis	Cucurbitaceae	Whole plant	Jazan Region [84]	The C. grandis extract (0.75 mg/kg, orally) showed remarkable glycemic effect which confirmed antidiabetic potential in streptozotocin-induced diabetic rats [103].
34	*Jatropha curcas*	Euphorbiaceae	Leaves	Jazan Region [84]	Ethanolic extract of *J. curcas* leaves at doses of (250 and 500 mg ml^−1^ bw by administered orally) reduced glucose level from 219.5 to 116.5 and 237 to 98.8, respectively, in alloxan-induced diabetic rats. The results were comparable to reduction in rats treated with the standard glibenclamide 232–94.5 at 600 *μ*g kg^−1^ [104].
35	*Ricinus communis*	Euphorbiaceae	Leaves	Jazan Region [84]	*R. communis* extracts at doses of 300 and 600 mg/kg/BW administered orally caused hyperglycemia in a dose-dependent manner in streptozotocin-induced diabetic rats [105].
36	*Ficus carica*	Moraceae	Leaves	Jazan Region [84]	A review article focusing on antidiabetic potential of *F. carica* confirmed that different extracts and fractions of *F. carica* and different doses significantly reducing hyperglycemia in streptozotocin-induced diabetic rats compared to standard drug [106].
37	*Ficus sycomorus*	Moraceae	Leaves	Jazan Region [84]	Alloxan-induced type 2 diabetic albino Wistar rats treated with 250, 500, and 1000 mg/kg (body weight) of the extract of *F. sycomorus* intraperitoneally reduced glucose level in diabetic rats almost to the normal as compared to diabetic control [107]
38	*Sesamum indicum*	Pedaliaceae	Seeds	Jazan Region [84]	Alloxan-induced diabetic rats treated with 5% and 10% of *Sesamum indicum* seed powder significantly decreased blood glucose and increased insulin levels as compared with the positive (diabetic) control group [108]
39	*Plantago ovata*	Plantaginaceae	Husk	Northern border region of Saudi Arabia [18]	In intravenous administration of alloxan-induced diabetic rabbits glucose level lowering effect observed (time dependent manner) with *P. ovata* husk extract of dose level (300 mg/kg, orally administered) [109]
40	*Polygala erioptera*	Polygalaceae	Aerial part	Jazan Region [84]	0.7 g/kg of *P. erioptera* extract showed significant antidiabetic effect compared to standard drug metformin and glibenclamide in normal and alloxan-induced diabetic rats [110]
41	*Polygonum aviculare* L	Polygonaceae	Aerial parts	Taif Region [111, 112]	Many ethnopharmacological investigations reported its antidiabetic potential but still need to study its in vivo and in vitro antidiabetic potential [113, 114]
42	Ziziphus *spina-christi*	Rhamnaceae	Leaves	Eastern region of Saudi Arabia [84, 112]	The strongest (*P* < 0.001) antidiabetic activity (25.59 and 39.48% after 7 and 15 days, respectively) was found following treatment with dose level of 500 mg/kg of *Z. spina-christi* extract in streptozotocin-induced diabetes mice [115].
43	*Bacopa monnieri*	Scrophulariaceae	Aerial parts	Jazan Region [84]	*B. monnieri* extract at dose level of 50, 100, 200, and 400 mg*/kg* significantly inhibited (33.3, 34.2, 42.1, and 44.2%, respectively) the increase in serum glucose concentration in a dose-dependent manner compared to standard drug [116].
44	*Lycium shawii*	Solanaceae	Aerial parts	Taif Region [112]	The strongest (*P* < 0.001) antidiabetic activity of *L. shawii* extract of 250 and 500 mg/kg bw was found in a dose-dependent manner in streptozotocin-induced diabetes rats [117].
45	*Solanum nigrum*	Solanaceae	Whole plant	Jazan Region [84]	*S. nigrum* extract was given orally in the dose level of 200 and 400 mg/kg/day (7 days) significantly lowering the blood glucose level in fasting compared to standard drug in alloxan-induced diabetic albino Wistar rats [118].
46	*Withania somnifera*	Solanaceae	Leaves	Jazan Region [84]	*W. somnifera* extract oral administration at two doses (200 and 400 mg/kg) reduced the blood glucose level significantly (*P* < 0.001) in a dose‐dependent manner in streptozotocin-induced diabetes rats. Only WS treatment did not register any significant change in the blood glucose level when compared to citrate control rats [119]. Another study also confirmed similar results in alloxan-induced diabetic rats [120]
47	*Lantana camara*	Verbenaceae	Leaves	Jazan Region [84]	Literature survey showed that *L. camara* leaf extract oral administration (200, 250, and 500 mg/kg of bw) showed antidiabetic potential in alloxan-induced diabetic rats [121]
48	*Peganum harmala*	Zygophyllaceae	Seeds	Taif Region [112]	*P. harmala* seed extract at dose level of (30, 60, and 120 mg/kg, orally administered for four weeks) significantly decreases in blood glucose (in all doses, *P* < 0.001), in comparison with diabetic group [122].
49	*Tribulus terrestris*	Zygophyllaceae	Stem, leaves	Jazan Region [84] Taif Region [112]	*T. terrestris* extract at (2 g/kg body weight) produced protective effect in streptozotocin-induced diabetic rats by inhibiting oxidative stress [123]. T. terrestris L. extract (250 mg/kg of bw orally administered) significantly lowers glucose level to normal compared to standard drug in glucose-loaded normal rabbits [124]
50	*Urtica dioica*	Urticaceae	Leaves	Wild plant, Tanhat, Saudi Arabia [125]	Urtica dioica extract at 100 mg/kg (*P* < 0.01) and 200 mg/kg (*P* < 0.001) significantly decreased serum glucose fructose-induced insulin resistance rats [126]. The aqueous extract of *U. dioica* significantly (*P* < 0.001; 67.92%) reduced the blood glucose level at dose of 300 mg/kg, IP) in streptozotocin-induced diabetes rats [127]

**Table 2:** Active ingredients and toxicological evaluation of the medicinal plants given in Table 1.

S. No	Names	Active ingredients	Toxicological evaluation
1	*Allium cepa*	Quercetin, N-acetylcysteine, alliuocide, cycloalliin, S-methyl-L-cysteine, S-propyl-L-cysteine, sulfoxide, dimethyl trisulfide, S-methyl-L-cysteine sulfoxide [128]	The animals tested were found healthy with no sign of toxicity up to the dose of 2 500 mg/kg. However, at 5 000 mg/kg, animals were weak and had intense extreme tachycardia and disorientation but no death was recorded. Thus, LD_50_ was more than 5 000 mg/kg [129].
2	*Anthemis herba alba*	Guainalides, eudesmanolide, pseudogua inolides, xanthonolides, flavone, flavonol glycosides, hispidulin, cirsilineol, vicenin-2, schaftoside, isoschaftoside, 5′,4-dihydroxy-6,7,3-trimethoxyflavone, quercetin-3-rutinoside, patuletin 3-rutinoside, patuletin 3-glucoside [130]	The available toxicological investigations have shown generally that *Anthemis herba-alba* is free from toxic effects at the different doses used in the studies [130]
3	*Cichorium intybus*	Chicoric acid, inulin, cichoralexin, cichoriin, esculetin, isochlorogenic acid, chlorogenic acid, caffeic acid, dicaffeoylquinic acid, aesculin, arginine, histidine, isoleucine, leucine, lysine, methionine, cysteine, phenylalanine, tyrosine, threonine, valine, serine, glutamic acid, glycine, alanine, aspartic acid, and proline [44, 45]	There were no treatment-related toxic effects from chicory extract administered orally at 70, 350, or 1000 mg/kg/day. There were no observed adverse effects of chicory extract in these studies [45]
4	*Clitoria ternatea*	Kaempferol, quercetin, myricetin, taxaxerol, tannic acid, 3-monoglucoside, *β*-sitosterol, delphinidin-3,5-diglucoside, anthoxanthin glucoside, p-hydroxycinnamic acid, kaempferol 3-neohesperidoside, myricetin 3-rutinoside, hexacosanol [48]	Ethanolic extract of aerial parts and root of CT led to lethargy in mice at the doses of 1500 mg/kg and above, orally [31]. Ptosis was seen above 2000 mg/kg dose in mice. Through intraperitoneal route, 2900 mg/kg dose was lethal within 6 hr due to severe CNS depression [47].
5	*Ficus carica*	Over 100 bioactive compounds have been identified in fig such as rutin, arabinose, chlorogenic acid, *β*-amyrins, syringic acid, *β*-carotenes, glycosides, *β*-sitosterols, and xanthotoxol [131]	The rats tested were found healthy with no sign of toxicity up to the dose of 5000, 5500, and 6000 mg/kg. However, at 5 000 mg/kg, animals were weak and had intense extreme tachycardia and disorientation but no death was recorded. Thus, LD_50_ was more than 6000 mg/kg [132]
6	Ficus benghalensis	Leucopelargonidin-3-0-*α*-L rhamnoside, eucodelphinidin, leucoanthocyanidia, leucoanthocyanin, *α*-amyrin acetate [53]	In acute toxicity studies, no mortality and signs of toxicity were observed at the dose of 2000 and 5000 mg/kg body weight for aqueous and ethanol extracts, respectively [53]
7	*Ficus religiosa*	Lupeol, *β*-sitosterol, *β*-sitosterol-d-glucoside, stigmasterol, lanosterol, campesterol, octacosanol, methyl oleonate, lupen-3-one, bergapten, and bergaptol [53]	Acute toxicity reported up to dose level 2000 mg/kg showed no mortality [53]
8	*Ficus microcarpa*	Polyphenols, organic acids, alkaloids, polysaccharides, megastigmanes, pheophytins, catechin, epicatechin, isovitexin, phenolic acids [53]	The oral administration of a single dose of 2000 mg/kg ethanol or methanol extract of leaves showed no mortality or behavioral alterations in the tested animals [53]
9	*Hypericum perforatum*	Quercitrin, rutin, hypericin, kaempferol, biapigenin, hyperforin [133]	Acute toxicity studies revealed the nontoxic nature of the H. perforatum [55]
10	*Anethum graveolens*	Carvone, *α*-phellandrene, limonene, dill ether, myristicin coumarins, flavonoids, phenolic acids, steroids [134]	The mice treated with AG of different doses of 1000, 2000, 3000, 4000, and 5000 mg/kg of body showed no toxicity [135]
11	*Cuminum cyminum*	Cuminaldehyde, limonene, *α*- and *β*-pinene, 1, 8-cineole, o-and p-cymene, *α*- and *γ*-terpinene, safranal, and linalool [58, 59]	The acute lethal toxicity test revealed that cumin crude extract was very safe [58]
12	*Marrubium vulgare*	Furanic labdane diterpenes, marrubenol, marrubiin, ladanein [60]	An acute toxicity study of *M. vulgare* (1 g/kg) extract orally administered at a dose of 1 g/kg body weight to the mice and treated mice showed tachycardia 1 h after intake of the infusion and loss of appetite 3 h after intake of the infusion. In another experiment, a single dose of 2000 mg/kg extract of M. vulgare for an acute toxicity study showed no toxicity [60].
13	*Mentha longifolia*	Lucenin-1, lucenin-2, camphelinone, camphene, carveol, carvone, carvone oxide, limonene, linalool, menthatriene, menthofuran, menthol, menthone, myrcene, p-cymene, piperitenone, piperitone, sabinene, *α*-pinene, *α*-terpinene, *α*-terpineol, longifone, pulegone, longifoamide-A, longifoamide-B, longiside-A, longiside-B, eugenol, salvianolic acid, eriodictyol-7-rutinoside, apigenin-7-O-glucoside, hypolaetin, longitin, luteolin, etc. [136]	*M. longifolia* extract was safe, and no toxicity or mortality was observed in both the oral (3200 mg/kg) and intraperitoneal (1730 mg/kg) administration in rats. Fourteen days of oral administration of the essential oil (125, 250, 375, and 500 *µ*L/kg) resulted in the reduction of red blood cells and lymphocytes and elevation of neutrophils and monocytes compared with normal animals [136].
14	*Origanum syriacum*	Carvacrol, thymol, thymoquinone [137]	Not available
15	*Teucrium oliverianum*	8-O-acetylharpagide, 12-O-methylteucrolin A, teucrolivin A, eupatorin, teucrolivin B, *μ*24(S)-stigmasta-5,22,25-trin-3*β*-ol [66]	Not available
16	*Teucrium polium*	Apigenin, luteolin, rutin, cirsiliol, cirsimaritin, salvigenin, and eupatorin in the roots, aerial parts, and inflorescences, teucardoside, b-sitosterol, stigmasterol, campesterol, brassicasterol, and clerosterol [68]	All rats treated with different concentrations of the total extract of TP were alive during the 14 days of observation. The animals did not show visible signs of acute toxicity. It suggested that the LD50 of the total extract was higher than 8 g/kg [138]
17	*Achyranthes aspera*	Aliphatic acid, betaine, achyranthine, *β*-ecdysterone, achyranthes saponins A, B, C, D, oleonolic acid, glycosides, triacontanol, E-sitosterol and spinasterol, triacontanol, hydroquinone, eugenol [70]	In acute oral toxicity studies, there was no increase or decrease in any of the parameters studied, in comparison with control animals [139]
18	*Aerva lanata*	Quercetin, betulin, aervine, ervoside, methylervine, aervine, lupeol, kaempferol, aervolanine, aervolanine, ervoside, methylaervine, persinosides A and B, tannic acid, lupeol acetate, benzoic acid, methyl grevillate [140]	The LD_50_ of the extract of AL for oral and IP acute toxicity tests were 22.62 g/kg and 0.432 g/kg, respectively. The extract produced apparent changes in body weights of both male and female rats and increased the weights of lung, brain, and pancreas of female rats while reducing the weight of testes in male rats. Hematological parameters were also altered [72]
19	*Alternanthera sessilis*	Stigmasterol, *β*-sitosterol, *β*-carotene, ricinoleic acid, myristic, palmitic, stearic, oleic, and linoleic acids, *α*-spiraterol, uronic acid, cyclo eucalenol, choline, oleanolic acid, lupeol [141]	The crude extract did not show any toxicity in mice even at the highest dose tested [75]
20	*Carissa edulis*	Β-Amyrin, (+)-carissone, 2*α*-carissanol, 6*α*-carissanol, dehydrocarissone, pinene, myrcene, limonene, sabanene, rutin, epicatechin gallate, carinol, lariciresinol, *β*-sitosterol, sitosterol glucoside, stigmasterol glucoside, scopoletin, isofraxidin, pinitol [77]	Lethal effects were not observed after the oral administration of the standardized ethanol extract at doses of 1600, 2900, and 5000 mg/kg. No behavioral changes were observed during the observation period. The oral LD50 of the extract was estimated to be greater than 5000 mg/kg. [142].
21	*Catharanthus roseus*	Vinblastine, vincristine, vindesine, vindeline tabersonine, ajmalicine, vinceine, vineamine, raubasin, reserpine, catharanthine, rosindin [143]	No mortality, but dose level higher than 300 mg of *C. roseus* extract can produce signs of biochemical and histopathological toxicity in liver, kidney, and heart. It is recommended that lower doses than the studied ones should be used for treatment [144]
22	*Rhazya stricta*	Polyneuridine, stemmadenine, strictanol, rhazimine, rhazinilam, rhazimanine, sewarine, vallesiachotamine, tetrahydrosecamine [145]	Daily oral dosing of *R. stricta* extract (0.25 g/kg) for 42 days was not fatal to sheep [145].
23	*Calotropis procera*	Calotropin, calotoxin, calactin, uscharin, voruscharin, uzarigenin, syriogenin, proceroside, calotropagenin, calotropain enzymes, *α*-amyrin, *β*-amyrin, lupeol, *β*-sitosterol, ursolic acid, calotropin, gigantin, giganteol [146]	2000 mg/kg body weight in single oral administration of aqueous and hydroalcoholic extract did not cause any death after 72 h post-treatment in male and female mice. Daily administration of aqueous extract to male and female Wistar rats during 3 and 6 weeks at the dose of 20 mg/kg/day induced no mortality in either sex [147]. Whoever C. procera is a toxic plant that is avoided by grazing animals. Its latex is used by tribes to poison arrows used for hunting. If in contact with human eye, it could cause ocular toxicity, causing loss of vision and photophobia [146]
24	*Opuntia dillenii*	Betanin, betanidin, kaempferol, kaempferide, quercetin, isorhamnetin, *β*-sitosterol, C29-5*β*-sterols, taraxerol, friedelin, methyl linoleate, 7-oxositosterol, 6*β*-hydroxystigmast-4-ene-3-one, daucosterol, methyl eucomate, eucomic acid [85]	During the oral toxicity study of the crude drug in rats, given doses up to 50 ml/kg exhibited no symptoms of toxicity [85]
25	*Opuntia ficus indica*	Quercetin, isorhamnetin, kaempferol, luteolin, isorhamnetin, isorhamnetin glycosides, gallic acid, coumaric, narcissin, rutin, nicotiflorin, isoquercetin, ferulic acid [87].	In vivo toxicity study suggests that the oral administration of *Opuntia ficus indica* extract at levels up to 2000 mg/kg/day does not cause adverse effects in male and female rats [148].
26	*Capparis decidua*	n-Triacontane, n-pentacosane, *β*-carotene, n-triacontanol, kaempferol, quercetin, isodulcite, nanocosane, capric acid, glucocapparin, capparine, capparinine, cappariline, codonocarpin, *β*-sitosterol [88]	The oral administration of *C. decidua* extract (500, 1000, 2000, and 4000 mg/kg) did not provoke any gross behavioral changes or manifestations of toxic symptoms in male rats [149].
27	*Beta vulgaris*	Betaine, betacyanins, betaxanthins, oxalic acid, and ascorbic acid [89]	In acute oral toxicity studies, the BVBF did not show any sign and symptoms of toxicity and mortality up to 2000 mg/kg dose, considered relatively safe [150]
28	*Haloxylon salicorlicum Bunge*	Kaempferol, quercetin, betaine chloride, piperidine, anabasine, aldotripiperideine, haloxine, halosaline, oxedrine, tyramine, N-methyltyramine, scopoletin, scopolin, umbelliferone, xanthotoxol, isooxyimperatorin, esculetin, *β*-sitosterol, ursolic acid, *β*-amyrin [93].	*H. salicorlicum* extract at doses 0.1, 0.2, 0.3, 0.4, and 0.5 mL/kg orally administered in rats was safe and showed no mortality or adverse effect [92].
29	*Evolvulus alsinoides*	*β*-Sitosterol, betaine, shankpushpin, evolvine, caffeic acid, 6-methoxy-7-O-*β*-glucopyranoside coumarin, 2-C-methyl erythritol, kaempferol-7-O-*β*-glucopyranoside, kaempferol-3-O-*β*-glucopyranoside, quercetin-3-O-*β*-glucopyranoside, scopoletin, scopolin [151]	The Evolvulus alsinoides extract did not cause any mortality up to a dose of 1500 mg/kg body weight and no behavioral, neurological, and autonomic profiles and was found to be safe [151].
30	*Ipomea aquatica*	Caffeic acid, chlorogenic acid, quercetin glucoside, quercetin malonyl glucoside, quercetin diglucoside, catechin, isochlorogenic acid A, C, aspartic acid, glycine, alanine and leucine, 7-O-*β*-D-glucopyranosyldihydroquercetin-3-O-*α*-D-glucopyranoside [97, 98]	In acute toxicity studies, *I. aquatica* extract was found to be safe up to 2g to 5 g/kg in mice. No mortality or toxic symptoms were observed during the entire duration of the study [152, 153].
31	*Citrullus colocynthis*	Cucurbitane, gallic acid, kaempferol, cucurbitacin A-E, I-L, chlorogenic acid, caffeic acid, colocynthoside A,B,C; choline, almitic acid, stearic acid, linoleic acid, oleic acids, catechin, myricetin, *α*-tocopherol, *γ*-tocopherol, *β*-carotene [153]	*C. colocynthis* plant is safe to use. Studies showed that lethal dose (LD_50_) to be 200 mg/kg, which indicate that the studied plant is not toxic when comparing the LD_50_ values of most bioactive pharmaceuticals currently used in therapeutics [154]
32	*Citrullus lanatus*	Lycopene, vitamin A, cucurbitacin E, citrulline arginine, glutamine and aspartic acid, pectin, vitamin b-complex and minerals [155, 156]	In acute toxicity study, there was no mortality observed up to the maximum dose level of 2000 mg/kg body weight of the extract after administered orally [102]
33	Coccinia grandis	Cephalandrol, *β*-sitosterol, cephalandrins A and B, *β*-amyrin acetate, lupeol, cucurbitacin B, taraxerone, taraxerol, *β*-carotene, lycopene, cryptoxanthin, xyloglucan, carotenoids, *β*-sitosterol, stigma-7-en-3-one, lupeol, *β*-amyrin, *β*-sitosterol, taraxerol [157]	The acute toxicity study indicated that treatment of *C. grandis* is safe up to 2 g/kg tested on animal [158].
34	*Jatropha curcas*	Jatrophol, jatropha factor C1, C2, C3,C4, C5, C6, jatropholones A, B, palmarumycin CP1, JC1, JCV2, curcin, curcacycline A, curcain, *β*-amyrin, *β*-sitosterol, stigmasterol, friedelin, taraxasterol, diamide, pyrimidine-2,4-dione, nobiletin, tomentin [103]	Many researchers have confirmed that *J. curcas* is highly toxic for animal as well as human. All parts of *J. curcas* are toxic and toxic compound reported from this plant like lectins, curcin, phorbol esters, phytate, protease inhibitors [103]
35	*Ricinus communis*	Rutin, quercetin, gallic acid, ricin, ricin A, kaempferol-3-O-*β* rutinoside, gentistic acid, linolenic acid, *α*-pinene, *α*-thujone, stigmasterol, ricinine, *β*-sitosterol, lupeol, castor oil [159, 160]	*R. communis* extracts given by oral route were safe up to a dose of 2,000 mg/kg/BW and did not show any mortality and toxic effects in the behavior of the treated animals [104].
36	*Ficus carica*	Ferulic acid, quercetin-3-O-glucoside, quercetin-3-O-rutinoside, psoralen, bergapten, coumarin, oleanolic acid, eugenol, angelicin, germacrene D, menthol, *α*-pinene, *β*-pinene [161]	Toxicity of 70% methanolic extract of Ficus carica leaves showed LD_50_ value of brine shrimp assay was 0.158 mg/ml [162]
37	*Ficus sycomorus*	Tannins, saponins, flavones, aglycones, anthraquinone glycosides, and flavonoid glycosides [53]	*F. sycomorus* methanol extract of stem bark is nontoxic up to the dose of 5000 mg/kg [53]
38	*Sesamum indicum*	Thelignans, sesamolin, sesamin, pinoresinol, lariciresinol, *α*-globulin, *β*-globulin, triacylglycerols, oleic, linoleic acids, sesamol, *γ*-tocopherol, 2-furfurylthiol, 2-phenylethylthiol, 2-methoxyphenol, 2-pentylpyridine, vitamin E, quinone, sesangolin [163, 164]	*S. indicum* ethanol extract is safe, up to dose level of 2000 mg/kg in acute toxicity studies in tested animals [165]
39	*Plantago ovata*	Hemicellulose,d-xylose, l-arabinose, d-glucose, d-galactose, and l-rhamnose, 5, 6,8-epiloganic acid, gardoside, plantamajoside [166]	4,5 Gram seed husk one to four times a day soaked in 150 ml of warm water recommend by WHO. Studies confirmed its side effect like bloating, gas, and allergy. No mortality reported [167]
40	*Polygala erioptera*	Helioxanthin [168]. No literature available. Recommended for natural products, isolation, biological and toxicological evaluation.	No literature available. Recommended for pharmacological and toxicological evaluation.
41	*Polygonum aviculare* L	Protocatechuic acid, catechin, myricitrin, epicatechin-3-O-gallate, avicularin, quercetin, juglanin, kaempferol, myricetin 3-0-(3′-0-galloyl-rhamnopyranoside, cinaroside, liquiritin, rutin [169, 170]	No data available
42	Ziziphus *spina-christi*	Jujuboside B1, christinin A, christinin A1 and A2, lotoside II, catechin, epicatechin, kaempferol 3-O-(6-O-rhamnosyl-galactoside), quercetin 7-O-(6-O-rhamnosyl-glucoside), quercetin 3-O-glucoside, kaempferol 3-O-glucoside [171, 172]	Butanol and water extract of Ziziphus *spina-christi* up to 100 mg/kg and 5 g/kg, respectively, in animal model produced no functional or structural disturbances in liver and kidney and no hematological changes [173, 174]
43	*Bacopa monnieri*	Bromine, *β*-sitosterol, betulinic acid, stigmasterol nicotinine, herpestine, bacosides A, bacopasides (I, II, III, IV, V), pseudojujubogenin glycoside, saponins (A, B, C) [175]	B. monnieri extract at the dose of 5,000 mg/kg did not cause any side effects. Similarly doses of 30, 60, 300, and 1,500 mg/kg given for 270 days did not produce any toxicity in rats [176].
44	*Lycium shawii*	Lyciumate, cyclopentapyrrolidine, imidazole, piperidine, nortropane, tropane, pyrrole, spermine, costunolide, catechin, lyciumaside, emodin, betulinic acid, *β*-sitosterol glucopyranoside, quercetin, gallic acid, rutin, *ρ*-coumaric acid, ferulic acid [177, 178]	Reported data revealed that LD_50_ of the *L. shawii* extract was greater than 2000 mg/kg b.w in animal model [179].
45	*Solanum nigrum*	Chlorogenic acid, quercetin, naringenin, solasodine, solamargine, solasonine, *α*-solanigrine, *β*-solanigrine, ascorbic acid, nigrumnins I and II [180]	*S. nigrum* extract at a dose of 2000 mg/kg p.o. was safe and showed no changes/alteration in normal behavior in animal model. No mortality was observed [181]
46	*Withania somnifera*	Withanolides, withaferin, withaferin A, withanone, withanolide A, withanolide IV, withanolide V, withanolide D [182].	LD_50_*value of W. somnifera* extract in rates was greater than 2000 mg/kg body weight. Compared to the control group in subacute toxicity study, administration of extract did not show any toxicologically significant treatment-related changes in clinical observations, and the toxicological studies revealed that the reasonable doses of W. somnifera are nontoxic and safe [182, 183]
47	*Lantana camara*	Eicosane, squalene, *β*-ionone, caryophyllene oxide, *β*-caryophyllene, hexanoic acid, tiglic acid, lantanilic acid, camaric acid, lantadene B, oleanolic acid, lantadene A, lantaninilic acid, lantoic acid, ursolic acid, betulinic acid [184]	*L. camara* extracts at dose level of 2000 mg/kg and 5000 mg.kg body weight in mice and rats, respectively, showed no significant toxic signs or mortality [185, 186]
48	*Peganum harmala*	Harmine, harmaline, harmalol, harman, vasicine and vasicinone, pegamine, acacetin 7-O-rhamnoside, 7-O-6″-O-glucosyl-2 ″-O-(3‴-acetylrhamnosyl) glucoside, 7-0-(2‴-0-rhamnosyl-2″-O-glucosylglucoside), peganone 1 and 2, *p*-cymene, limonene, eugenol, thujico acid, *β*-cubebene [187–189]	*P. harmala* different doses of different extracts in animal and human clinical studies confirmed that this plant showed side effect like intoxications, abdominal writhing, body tremors, and toxic at high does level causing paralysis, liver degeneration, euphoria, convulsions, nausea, vomiting, hypothermia. However, therapeutic doses have been reported to be safe in a rodent model [189]
49	*Tribulus terrestris*	Naringin, rutin, hyperoside, quercitrin, naringenin, quercetin, hesperetin, kaempferol, apigenin, pyrogallol, gallic acid, catechin, catechol, chlorogenic acid, caffeic acid, vanillic acid, ferulic acid, salicylic acid, ellagic acid, coumaric acid, cinnamic acid [190]	*T. terrestris* extract showed no mortality/or toxicity at a dose up to 1 g kg^−1^ of bw in mice [190]
50	*Urtica dioica*	*β*-Amyrin, *β*-sitosterol, stigmasterol, oleanolic acid, ursolic acid, quercetin, rutin, chlorogenic, and 2-O-caffeoyl malic acid expressed as caffeic acid, isoquercetin, kaempferol 3-O-rutinoside [191–193].	*U. dioica* extracts up to dose level of 2000 mg/kg body weight in animal model showed no mortality or changes/alteration in normal behavior [127].

**Table 3:** Selected antidiabetic phytoconstituents and its targeted metabolic pathway.

Phytoconstituents	Targeted metabolic pathways	Structures
Catharanthine	Free radical; our body has a defense system containing several enzymes, which are catalase, superoxide dismutase, and glutathione-S transferases and reduced glutathione. Catharanthine activates these free radical scavenging enzymes and prevents our body from their adverse effects.	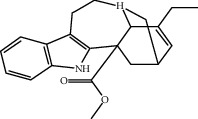
Harmine	Insulin secretion and *β*-cell regeneration	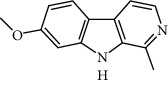
Betaine Achyranthine *β*-ecdysone	Carbohydrate digestion and absorption	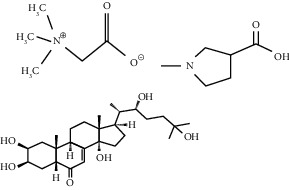
Apigenin	Cholesterol synthesis, glycogen synthesis	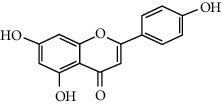
Betaine choline	Regeneration of pancreatic *β* cells and insulin secretion	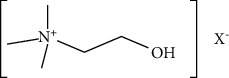
Ferulic acid	Free radical scavenging activity, insulin secretion	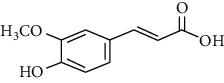
Leucine, isoleucine, alanine	Insulin secretion	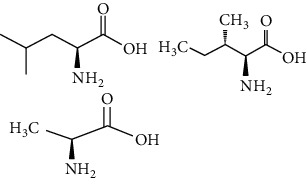
Chlorogenic acid	Krebs cycle	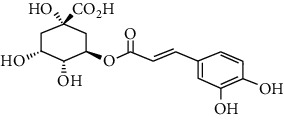
Emodin, cinnamic acid	Insulin secretion	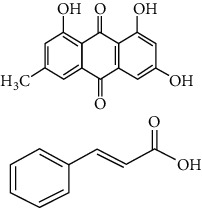
Eugenol, *α*-pinene, limonene, p-cymene thujone	Insulin secretion, regeneration of pancreatic *β* cells	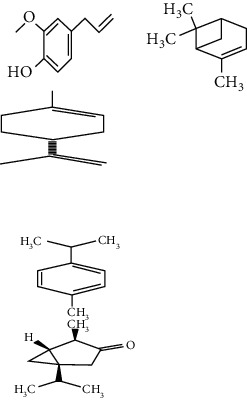
Pectin	Glucose transport, carbohydrate metabolism, stabilizing agents	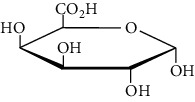
Inulin	Glucose transport, carbohydrate digestion and absorption	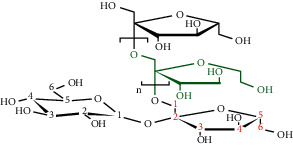
Cucurbitacin B	Insulin secretion, glycogen synthesis	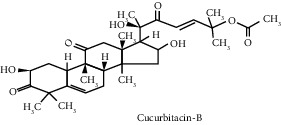
Catechin Epicatechin	Scavenging activity Insulinomematic	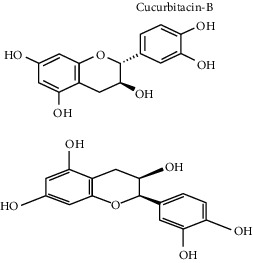
Naringin	Glycogen synthesis, glycolysis, gluconeogenesis	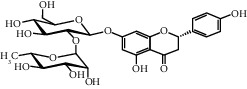
Flavones	Insulin secretion	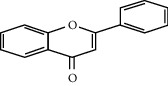
Quercetin, quercitrin, apigenin, rutin, apigenin-7-O-glucoside	Insulin secretion	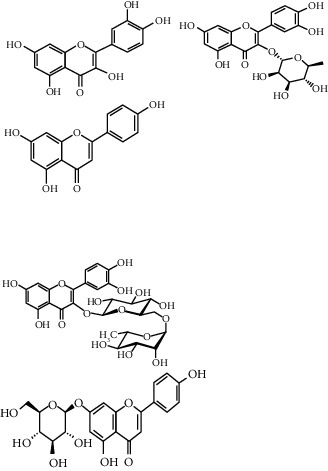
Naringenin	Insulin secretion	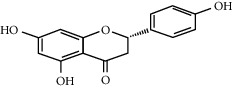
*α*-Terpineol	Insulin secretion	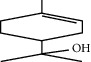
Kaempferol, isorhamnetin, caffeic acid, p-coumaric acid	Free radical scavenging activity Carbohydrate digestion and absorption, insulin secretion	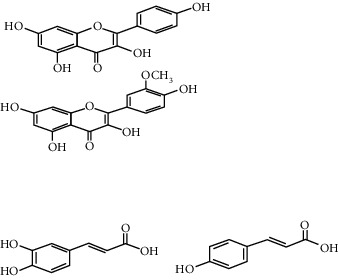
Vitamin A, E	Free radical scavenging activity	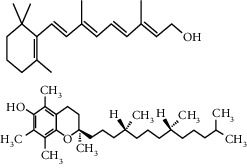
Ellagic acid	Carbohydrate digestion and absorption, insulin secretion	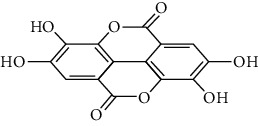
Carvacrol, linalool	Insulin secretion, carbohydrate digestion and absorption	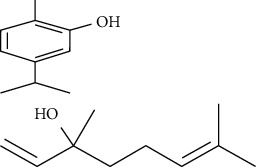
Stigmasterol	Regeneration of pancreatic *β* cells, insulin secretion	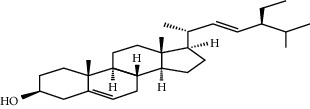
Ursolic acid	Regeneration of pancreatic *β* cells and insulin secretion	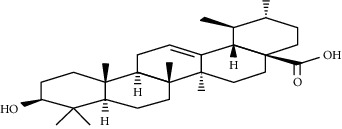
*β*-Sitosterol	Insulin receptor (IR) and glucose transporter 4	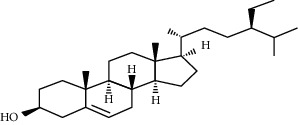

## Data Availability

This is a review article. All data are taken from published research papers and available online.
